# Putting the genie back in the bottle? Availability and presentation of oral artemisinin compounds at retail pharmacies in urban Dar-es-Salaam

**DOI:** 10.1186/1475-2875-5-25

**Published:** 2006-03-29

**Authors:** S Patrick Kachur, Carolyn Black, Salim Abdulla, Catherine Goodman

**Affiliations:** 1 Ifakara Health Research and Development Centre, Ifakara, P.O. Box 78373, Dar-es-Salaam, Tanzania; 2 United States Public Health Service Commissioned Corps and Malaria Branch, Division of Parasitic Diseases, National Center for Infectious Diseases, Centers for Disease Control and Prevention, Atlanta, Georgia, USA; 3 University of Maryland Baltimore County, Baltimore, Maryland, USA; 4 Health Economics and Financing Programme, Health Policy Unit, London School of Hygiene and Tropical Medicine, London, UK

## Abstract

**Background:**

Recently global health advocates have called for the introduction of artemisinin-containing antimalarial combination therapies to help curb the impact of drug-resistant malaria in Africa. Retail trade in artemisinin monotherapies could undermine efforts to restrict this class of medicines to more theoretically sound combination treatments.

**Methods:**

This paper describes a systematic search for artemisinin-containing products at a random sample of licensed pharmacies in Dar-es-Salaam, Tanzania in July 2005.

**Results:**

Nineteen different artemisinin-containing oral pharmaceutical products, including one co-formulated product, one co-packaged product, and 17 monotherapies were identified. All but one of the products were legally registered and samples of each product were obtained without a prescription. Packaging and labeling of the products seldom included local language or illustrated instructions for low-literate clients. Packaging and inserts compared reasonably well with standards recommended by the national regulatory authority with some important exceptions. Dosing instructions were inconsistent, and most recommended inadequate doses based on international standards. None of the monotherapy products mentioned potential benefits of combining the treatment with another antimalarial drug.

**Conclusion:**

The findings confirm the widespread availability of artemisinin monotherapies that led the World Health Organization to call for the voluntary withdrawal of these drugs in malaria-endemic countries. As the global public health community gathers resources to deploy artemisinin-containing combination therapies in Africa, planners should be mindful that these drugs will coexist with artemisinin monotherapies in an already well-established market place. In particular, regulatory authorities should be incorporated urgently into the process of planning for rational deployment of artemisinin-containing antimalarial combination therapies.

## Background

Artemisinin-containing combination treatments (ACTs) are recommended to stem the rising tide of drug-resistant malaria in Africa[1]. So far, widespread public-sector deployment of artemisinin-containing drugs has been constrained by their relatively high cost and limited global supply, as well as some lingering concerns about safety[2,3]. International attention has focused on identifying sustainable resources for purchasing the needed drugs for widescale deployment through public sector health systems[4,5]. However, artemisinin-containing compounds have been available in retail pharmacies in many African countries for some time. In Tanzania, no fewer than 31 artemisinin-containing compounds have been registered with the Food and Drugs Authority[6]. These include a range of oral and parenteral products sold from pharmacies as prescription only medicines. Parenteral preparations are specifically intended to be administered only by a trained health worker; however, oral products–even those registered as prescription only medicines–are frequently obtained without a prescription and require that the patient or a caretaker make a choice between several products and interpret the dosing instructions provided with the packaging[7]. Therefore, this study was undertaken to identify and describe the range of oral artemisinin products currently available in one urban setting.

No previously published studies report on the quality of packaging and labeling materials for artemisinin-containing medicines. Recent reports from Asia have raised questions about the quality of commercially available artemisinin products, but have been largely limited to details on chemical content and bioavailability[8]. The presentation of a pharmaceutical product can encourage or dissuade clients from selecting it. The quality of dosing instructions, including their appropriateness for low-literate clients can have an important impact on complete adherence. Therefore, the study aimed at describing qualitative aspects of presentation for these artemisinin-containing products as well as their availability. These findings were compared to national standards for the presentation of pharmaceutical products which have been established by the Tanzania Food and Drugs Authority (TFDA), alongside requirements for chemical content and bioavailability[9].

Finally, according to Tanzania's national malaria treatment guidelines artemisinin monotherapies are recommended only as alternative regimens for patients who cannot tolerate the first-, second- or third-line drugs[10]. The World Health Organization (WHO) recommends that this class of drugs be deployed only in combination with other antimalarial drugs as a safeguard against the development of unchecked resistance[11] and has called for a voluntary halt to marketing the monotherapies in malaria-endemic countries[12]. A number of national and global recommendations pertaining to the use of artemisinin-containing medicines have been elaborated since the first products in this class were registered[13]. This analysis also compared the manufacturers' recommended doses for these products with published international standards.

## Methods

Dar-es-Salaam is the commercial capital and largest major urban center in Tanzania. Home to more than three million people, it hosts a major Indian Ocean port and comprises three independently governed municipalities. Malaria transmission can occur throughout the year in Dar-es-Salaam, although the intensity of transmission in this urban setting is far lower than elsewhere in the country. None-the-less, malaria is perceived as a very common illness. It remains the single most common clinical diagnosis at health facilities in the municipalities [14]. In addition, people commonly perceive malaria to be the cause of nearly every uncomplicated febrile illness and routinely seek treatment without consulting health workers at registered facilities or obtaining a diagnosis[15].

Artemisinin-containing drugs are recognized as prescription only medications in Tanzania and can be stocked only at registered health facilities (health centres and hospitals with a licensed physician or clinical officer) and fully-licensed pharmacies (designated as Part I pharmacies). Thirty registered Part I pharmacies in the three municipalities were randomly selected from a line-list of 331 such shops provided by TFDA. Based on previous experience with similar activities in 2003 and 2004, the study team developed a questionnaire to assess the availability of artemisinin-containing antimalarial drugs at these shops during the first two weeks of July 2005. The survey instrument included scanned images of 18 products identified in previous years and asked pharmacists to identify any additional products in this class which were in stock at the time of visit or within the previous three months. The pharmacists provided the current retail price of each product they had recently stocked. They were also asked their opinions on which three drugs in this class they sold most frequently. A sample of each commercial product was purchased so that researchers could compare the packaging and labeling information. Samples were obtained without a prescription, but after explaining the purpose of the study to the pharmacist in charge.

Data from the survey were entered into EpiInfo Version 6.0 (Centers for Disease Control and Prevention, Atlanta, Georgia USA). All packaging and package inserts were scanned into a digital format and were coded and analyzed using AtlasTi version 4.2 (Scientific Software, Berlin, GERMANY). Elements of drug presentation were compared to nationally recognized standards for labeling and package inserts as elaborated by TFDA[9] and dosing recommendations were compared to standards established by WHO[11].

## Results

### Availability, origin, price and estimated sales

Data were successfully collected in 29 pharmacies. Six of the pharmacies selected from the line list could not be located so other Part 1 drug stores in the same neighborhoods were substituted. One additional pharmacy could not be located and no appropriate substitute was found. All shops had at least two artemisinin-containing products available on the day of the visit, and one shop had 16 different products on offer and had stocked two additional items in the previous three months. The median number of artemisinin-containing oral pharmaceutical products available at selected pharmacies was 13 (mean = 10.8). In all 19 different products were identified, not including parenteral or rectal preparations. All but two products were artemisinin monotherapies. The exceptions were product #1 a co-formulated tablet containing artemether/lumefantrine and product #2, co-packaged tablets of artesunate and mefloquine. Nine (31%) pharmacists indicated that they were out of stock on the day of interview for one or more artemisinin-containing products that they regularly carried within the past three months. There was no observable trend for individual brands or products and no product had been stocked out at more than two outlets. The 19 products available are listed in Table 1 below, which also compares the relative prevalence of each product and its mean purchase price as well as pharmacists' perceptions of the best selling brands

**Table 1:** Artemisin-containing oral pharmaceutical products obtained from registered pharmacies in Dar-es-Salaam, Tanzania, 2005

**Product/active ingredient(s)**	**Formulation**	Country of origin*	**2005 availability** ** (n = 29)**	**Named in the top 3 sellers** ** in 2005 (n = 28)**	**Mean selling ** **price 2005**^†^
1. artemether/lumefantrine	20/120 mg tablets, ***co-formulated***	Switzerland	21 (72.4%)	5 (17.9%)	$ 7.59
2. artesunate and mefloquine	200 mg and 250 mg tablets, ***co-packaged***	Switzerland	17 (58.6%)	0	$ 7.05
3. artesunate	50 mg tablets	China	6 (20.7%)	0	$ 4.62
4. artesunate	50 mg tablets	France	24 (82.8%)	7 (25.0%)	$ 5.48
5. artesunate	50 mg tablets	Switzerland	8 (27.6%)	0	$ 5.26
6. artesunate	50 mg tablets	Belgium	28 (96.6%)	3 (10.7%)	$ 3.53
7. artesunate	50 mg tablets	India	17 (58.6%)	0	$ 2.51
8. artesunate	100 mg tablets	Belgium	28 (96.6%)	28 (100%)	$ 4.99
9. artesunate	100 mg tablets	Tanzania	7 (24.1%)	1 (3.6%)	$ 2.19
10. artesunate	100 mg tablets	Tanzania	20 (69.0%)	6 (21.4%)	$ 1.92
11. artesunate	100 mg tablets	Kenya	2 (6.9%)	0	$ 2.31
12. artesunate	200 mg tablets	Switzerland	19 (65.5%)	1 (3.6%)	$ 6.72
13. artemether	40 mg tablets	India	8 (27.6% 0	0	$ 2.24
14. artemether	50 mg tablets	China	24 (82.8%)	15 (53.6%)	$ 4.74
15. artemether	180 mg/60 ml suspension	Belgium	18 (62.1%)	0	$ 4.65
16. artemether	300 mg/100 ml suspension	Belgium	3 (10.3%)	0	$ 5.75
17. dihydroartemisinin	60 mg tablets	Korea	18 (62.1%)	0	$ 4.36
18. dihydroartemisinin	60 mg tablets	China	25 (86.2%)	9 (35.7%)	$ 4.55
19. dihydroartemisinin	160 mg/80 ml suspension	China	20 (69.0%)	0	$ 4.18

*Country of origin refers to the address of the company holding registration for this product in Tanzania (with the exception of product 11 which was not registered in the country). In some cases drugs that are produced in Asia are packaged and distributed by pharmaceutical partners in Africa and Europe. This information was not consistently provided.

^†^On 15 July 2005 = US $1.00 = 1188 Tanzania shillings

Eleven of the products contained artesunate, but products containing artemether and dihydroartemisinin were also commonly encountered. At least one product containing each of these compounds was identified at more than 80% of outlets. All products but one (product #11) were registered with TFDA and sold under the labels of pharmaceutical producers in Europe (n = 9), Asia (n = 7) and Africa (n = 3 including the one unregistered product). One European company was the source of four different products, two artesunate tablets and two artemether suspensions. Another European company supplied three products, two artesunate tablets and one product containing co-packaged artesunate and mefloquine. One Asian company was the source of two products, both tablets and suspension containing dihydroartemisinin. All other products represented the registering company's sole product in the class. In all the 19 products identified were registered to or produced by 13 different manufacturers.

Prices ranged from 12,800 Tanzanian shillings (TSh) (US $10.77) for an adult dose of product #1 to 1,300 TSh ($1.09) for an adult dose of product #10. The co-formulated and co-packaged ACT products (products #1 and #2, respectively) fetched the highest prices. However, at least one artesunate monotherapy (product #12) was priced similarly. Most of the European and Chinese monotherapy products sold for 5000–6000 TSh (roughly $4.00 to $5.00) for one adult dose, while those from Indian or African manufacturers were considerably less expensive. Prices for individual products varied among the shops, but relative differences between specific products within a given shop were remarkably similar across all outlets. When asked to comment on the three best selling brands of artemisinin-containing drugs, all 28 pharmacists who responded named the same European brand of artesunate monotherapy (one pharmacist declined to estimate relative sales of individual products but stocked only products from this manufacturer). The distant second and third most commonly named products were Asian brands of artemether and dihydroartemisinin tablets, respectively.

### Features of drug presentation

All of the products were sold as prepackaged unit doses, usually sufficient for an average adult taking the manufacturer's recommended course of treatment. All were obtained in the manufacturers' original packaging. Tablets were sealed in blisters or foil sachets and powder for suspension was provided in tamper-proof bottles. Each product included the manufacturer's package insert and was contained in a professionally manufactured box with high quality printing using no fewer than two colors. None of the medicines in this class were sold as loose tablets dispensed from bulk containers. All but three products were identified as antimalarial drugs on the overpackaging, most typically in English. Only three products included this information in Swahili. Other languages including French, Italian, Spanish, Chinese, and Portuguese were more commonly represented. Seven products included an image of a mosquito on the box, which might have helped low-literate and non-European consumers identify them as malaria treatments. However, in at least two cases the image was so stylized as to be of limited use for this purpose.

Table 2 includes information about how well the products matched legally recognized standards for packaging and labeling of pharmaceutical products in Tanzania[9] Most of the products conformed reasonably well with many of the standards, but there were some notable exceptions. The nonproprietary name always appeared in much smaller type font than the commercial name, except in the case of the single generic product. Only six products had their Tanzanian registration number printed on the packaging, suggesting many were packed for sale in multiple countries. Two additional products were labeled with registration numbers from other countries, one each for Nigeria and Ecuador. Seven products identified a manufacturer other than the registrant with statements such as "Manufactured by Company X in Asia for Company Y in Europe." In particular, many of the European and African products did not mention any other partners that might have been responsible for some stages of the production including extraction and tablet formulation, although this is likely to have been the case for many. Information on contraindications, drug interactions and how to recognize and manage overdose was inconsistently provided. Fewer than half included a date of publication for the package insert. Five products were packaged with inserts prepared for two or more preparations of the same drug.

**Table 2:** Features of packaging and labeling for oral artemisinin-containing pharmaceutical products at pharmacies in Dar-es-Salaam, Tanzania

**Feature**	**Frequency**
General features	
Packaged and sold as a unit dose	19 (100%)
Package insert included	19 (100%)
Image of mosquito appears on package	7 (37%)

TFDA Package and labeling requirements	
International non-proprietary name included	19 (100%)
Non-proprietary name is printed larger or bolder*	0
Strength of active ingredient identified	19 (100%)
Name and address of registrant identified	17 (89%)
Name and address of manufacturer other than registrant identified	7 (37%)
Labeled "Keep out of reach of children"	10 (53%)
Lot or batch number	19 (100%)
Expiry date	19 (100%)
Date of manufacture	18 (95%)
TFDA Registration number	6 (32%)
	
TFDA Package insert requirements	
Indication (uncomplicated malaria)	19 (100%)
Dosage regimen	19 (100%)
Contraindications	12 (63%)
Side effects	19 (100%)
Drug interactions	11 (58%)
Precautions and warnings	18 (95%)
Symptoms and treatment of overdose	11 (58%)
Presentation (e.g. "12 tablets of 100 mg each")	19 (100%)
Storage instructions	13 (68%)
Shelf life	14 (74%)
Name and address of manufacturer other than registrant identified	6 (32%)
Date of publication of package insert	9 (47%)

Appropriateness of recommended dose	
Recommended total adult dose consistent with international standards**	4 (21%)
Recommended duration consistent with international standards***	5 (26%)
Mentions that non-immune patients may require additional doses	2 (13%)
Gives dosages for children	17 (89%)
Specifically formulated for children	4 (21%)

*Excludes one generic product.

**At least 16 mg/kg for artesunate, artemether or dihydroartemisinin monotherapies; 9 mg/kg artemether combined with 72 mg/kg lumefantrine; or 12 mg/kg artesunate combined with 32 mg/kg mefloquine.

***At least 7 days for artemisinin monotherapies, 6 doses of artemether/lumefantrine over 3 days, or 3 daily doses for artesunate + mefloquine.

### Appropriateness of recommended doses

The recommended doses for the co-formulated (product #1) and co-packaged (product #2) ACTs were consistent with international recommendations. The World Health Organization recommends that artemisinin monotherapy be used only when combination treatments are unavailable or a patient cannot tolerate another antimalarial drug[11] and has recently called on manufacturers to withdraw these products from markets in malaria-endemic countries[12]. In the past, WHO recommended that at least seven days of monotherapy and a total dose of 16 mg/kg (1120 mg for an adult) was warranted, even in partially immune adults[16]. One recent report among non-immune patients suggests that even this prolonged monotherapy may be suboptimal[17]. Investigators compared the total dose of artemisinin recommended for an adult and calculated the recommended pediatric dose for an 18 month old child weighing 15 kg based on information in the package inserts. Three products recommended no adult dose. The total adult artemisinin dose recommended among the other products ranged from 360 mg (product #17) to 1200 mg (products #5 and 12). Ten products recommended a total adult dose of 600 mg of artemisinin. The recommended dose for an 18 month old 15 kg child was similarly variable, ranging from 105 mg (product #11) to 450 mg (products #5 and 12) total artemisinin dose (At lease 240 mg would be required to comply with WHO recommendations for such a child). Four products recommended a total aretmisinin dose of 144 mg and five recommended 180 mg for a child in this age or weight group. Two products recommended no pediatric doses and included the comments: "Not recommended for children < 30 kg." (product #2, the co-packaged product) and "Below 35 kg.: As directed by physician" (product #10).

Aside from the total dose, the recommended duration of therapy was inadequate for most of the products identified, particularly the artemisinin monotherapies. Of the 17 monotherapies identified, only the three dihydroartemisinin products recommended a seven day course of treatment. At least two other products included a statement about non-immune patients requiring additional doses and an extended duration of therapy. None of the monotherapy products included any information about the advantages or disadvantages of combining treatment with other antimalarial drugs. Thirteen products recommended regimens based on single daily doses, often with a loading dose double what was recommended on subsequent days. Three products recommended two doses on the first day of treatment followed by daily doses thereafter (products #1, 12 and 13). Three artesunate monotherapy products recommended twice daily doses (products #3, 4 and 7).

All of the products were packaged in unit doses. But in most cases these were sufficient only for the substandard doses recommended by the manufacturers. To obtain a complete seven day treatment adult patients would have had to purchase at least two "unit doses" of all of the artemether and artesunate monotherapy products. Four products were specifically identified as pediatric preparations (a 50 mg. artesunate tablet, a suspension of dihydroartemisinin and two preparations of artemether suspension). According to WHO recommendations, the number of tablets or volume of suspension available for each of these four pediatric products would have been sufficient for children ranging from up to 9.6 kg for one product all the way to 21.4 kg for another. Additional packages would have to be purchased to treat larger children completely.

## Discussion

A recent report from the United States (US) Institute of Medicine (IOM) describes a twofold challenge: "to facilitate widespread use of artemisinins while, at the same time, to preserve their effectiveness for as long as possible[18]." In its report, the group advances its case for a global level subsidy combined with a coordinated world-wide effort to procure and deploy artemisinin compounds exclusively in combination therapies co-formulated with other antimalarial drugs. Among other proposed advantages, the IOM authors anticipate that this approach would empower the global community to dissuade participating manufacturers from producing and promoting artemisinin monotherapies. Assuming the resource and production constraints are overcome and the global procurement mechanism established within the next 5 years it is important to recognize that unsubsidized, dynamic retail markets for artemisinin monotherapies are already established–and thriving–particularly in urban centers. A recent plea from the World Health Organization to persuade manufacturers to withdraw monotherapies from markets in endemic countries is welcome, but cannot be effectively enforced without the cooperation of industry and regulatory officials[12].

The findings suggest that this challenge be addressed as urgently as possible. They confirm that artemisinin-containing oral pharmaceutical products are already widely available at pharmacies in urban Dar-es-Salaam and underscore the urgency with which WHO called for an immediate halt to their proliferation. The number and range of products described in this study were nearly identical in a similar activity completed a year prior (the 2004 survey identified 17 products). While the implementation of an ACT for first-line malaria treatment in the public sector has been delayed in Tanzania by the need for donor support and a global shortage of raw material for artemisinin production[19], relatively few of the pharmacies visited had stocked out of these drugs at any time in recent memory. In addition to their broad availability, the products ranged widely in price. Although some were priced well beyond the reach of most Tanzanian consumers, the most affordable items in this class were priced only 50% higher than European products containing sulfadoxine/pyrimethamine, the currently recommended first-line treatment for malaria.

While many of the national standards for packaging and labeling were met, most products were packaged for monotherapy and dosing instructions varied as much as four fold across the range of products. In addition, most products recommended doses that were insufficient based on international recommendations. An additional issue of concern with the presentation of these products is that the regulatory requirements assume that the drugs will be available by prescription only. For that reason they include a level of detail that is appropriate for a physician or pharmacist, but would be very difficult for even an English-literate lay person to interpret. Recognizing that for the most part antimalarial drugs are dispensed and purchased not by trained health professionals, but by ordinary consumers, WHO has recently produced specifications for prepacking antimalarial medications[20]. Their report also recommends that careful packaging should be coupled with information, education and communication efforts to optimize consumers' ability to adhere to recommended therapies and dosing regimens. Malaria control officials and regulatory authorities may wish to reconsider their current requirements in light of these new specifications, particularly in countries like Tanzania, where a new national treatment policy recommending ACT is about to be launched.

These observations are discouraging. However, it is important to note that in Tanzania this complex market for artemisinin-based drugs is still relatively contained within major urban centres. The availability of pharmacies and expensive medical products in rural areas, where most malaria transmission occurs, is far more limited. In rural Tanzania, for example, drug shops seldom promote antimalarials other than the recommended first- and second-line drugs [21]; artemisinin-containing treatments are found only occasionally[22]. Nevertheless, the findings point to the urgent need for malaria control officials and drugs regulatory authorities in malaria-endemic countries to plan for a coordinated and rational deployment of artemisinin-containing antimalarial drugs. In Tanzania, and a number of other countries, the Ministry of Health anticipates introducing ACTs for first-line treatment of uncomplicated malaria as early as 2006[23]. It is unlikely that the global subsidy as recommended by IOM will have materialized by that time and it is unrealistic to expect that established products will be retrenched without substantial intervention. But funds are available to make a highly subsidized ACT available at public sector health facilities which could discourage many clients from resorting to relatively expensive treatments at pharmacies. However, people will continue to rely on retail sector sources for their flexibility and convenience[21] and some of the products identified are approaching affordability and can be expected to become ever more widely available. Interventions to expand the availability of ACT for home and community management of malaria might offer one way to work actively with pharmaceutical retailers to achieve high coverage and minimize misuse of monotherapies[24], but such an approach is unlikely to be appropriate everywhere ACTs are used[25]. Improved packaging and presentation might also be employed to enhance the likelihood that those who obtain artemisinin-containing drugs complete the recommended dose[26].

No single solution will likely reverse the current trend as countries move quickly to roll out ACTs. Rather, malaria control officials and drugs regulatory authorities will have to negotiate solutions and develop adaptive practices if the twin goals of achieving broad access and safeguarding against resistance are to be feasible. Monitoring the availability and presentation of already registered products, as well as carefully considering applications for registering new products should be undertaken as soon as possible. Reconsidering packaging and labeling requirements so that they result in more useful information and are consistent with national and international guidelines would go a long way to improving the situation as well. It may be a long while before a global subsidy on ACTs achieves the goal of crowding out artemisinin monotherapy products–if such a development ever occurs. Engaging pharmaceutical producers and regulators in the process of rationally rolling out ACT to meet the needs of malaria-endemic countries where traditional antimalarial drugs have become ineffective may be a more constructive way forward in the short- to medium-term.

## Conclusion

Artemisinin-containing monotherapies are already widely available in retail pharmacies in Tanzania and elsewhere, even while public health officials struggle to introduce more rational ACTs and global authorities have called for the recall of single drug treatments in this class. Packaging and labeling of these commercially available products is highly variable and manufacturers generally recommend insufficient doses and duration of therapy based on international recommendations. National and international plans to deploy ACTs should urgently engage pharmaceutical manufacturers and drugs regulatory authorities or risk undermining the long term utility of this entire class of drugs.

## Authors' contributions

SPK developed the research plan, selected the sample, supervised data collection, completed the analysis, and drafted the manuscript. CB adapted the study instruments, collected the samples, coded the data and contributed to this manuscript. SA contributed to the research plan and manuscript. CG conceived the research question and contributed to the research plan, data coding, analysis and manuscript. All authors read and approved the final manuscript.
